# A Case of Invasive Gastrointestinal Mycotypha Infection in a Patient with Neutropenia

**DOI:** 10.1155/2018/5864175

**Published:** 2018-09-02

**Authors:** Polina Trachuk, Wendy A. Szymczak, Peter Muscarella, Uzma N. Sarwar

**Affiliations:** ^1^Division of Infectious Diseases, Department of Medicine, Albert Einstein College of Medicine, Montefiore Medical Center, Bronx, New York, USA; ^2^Department of Pathology, Albert Einstein College of Medicine, Montefiore Medical Center, Bronx, New York, USA; ^3^Division of General Surgery, Department of Surgery, Albert Einstein College of Medicine, Montefiore Medical Center, Bronx, New York, USA

## Abstract

Gastrointestinal mucormycosis is a rare life-threatening infection to which neutropenic patients are especially vulnerable. *Mycotypha microspora* is a mucormycete that has not been described as a human pathogen. We discuss the successful eradication of gastrointestinal *Mycotypha microspora* in a neutropenic patient with simultaneous pulmonary *Aspergillus fumigatus* infection.

## 1. Introduction

*Mycotypha microspora* is a filamentous fungus first described as a genus in 1932 when it was noted to be a pathogenic organism of an orange [1]. A member of the order Mucorales, it has subsequently been reported as being isolated from decaying wood, a washroom of a hospital, and from clinical wound and stool samples where colonization was suspected [2, 3]. To our knowledge, it has never been described as a pathogen implicated in human infection. Furthermore, dual infection with *Aspergillus* and a mucormycete has only been described once, resulting in a fatality [4]. In this report, we discuss the treatment of a patient with a dual *Aspergillus fumigatus* and *Mycotypha microspora* infection.

## 2. Case Description

A 41-year-old white man presented to Montefiore Medical Center on June 15, 2017 with one day of fever and confusion. His past medical history was significant for recently diagnosed mediastinal germ cell tumor being treated with etoposide, ifosfamide, and cisplatin therapy. His last dose of chemotherapy was administered the week prior to presentation.

On presentation to the emergency department, he was febrile, hypotensive, tachycardic, and tachypneic. His initial white blood cell count was 0.1 with an absolute neutrophil count of zero. His creatinine was 5.32 mg/dL, elevated from a baseline of 2.0 mg/dL a week prior. A computed tomography (CT) of the chest, abdomen, and pelvis showed patchy bilateral airspace consolidations compatible with pneumonia. He was intubated, started on broad spectrum antibiotics, and admitted to the medical intensive care unit on multiple vasopressors for hemodynamic support. His clinical course was complicated by a progressive decline in hemoglobin associated with gastrointestinal (GI) bleeding that remained refractory to blood transfusions. Three endoscopies were performed that demonstrated gastric mucosal ischemia, multiple ulcers, and large quantities of blood in the upper GI tract. No active bleeding was identified. To identify the source of hemorrhage, the patient underwent a CT angiogram of the abdomen and a mesenteric angiogram, which were also unremarkable. Empiric embolization of the left gastric artery was performed with no resolution of bleeding. On hospital day 24, he developed a catastrophic upper gastrointestinal bleed requiring multiple transfusions of packed red blood cells. He underwent an emergent total gastrectomy for presumed stress gastritis. The stomach was grossly distended and filled with fresh blood. The patient was stabilized and returned to the operating room on postoperative day number two for reconstruction with a Roux-en-Y esophagojejunostomy, feeding jejunostomy tube placement, and formal abdominal wall closure.

**Figure 1:**
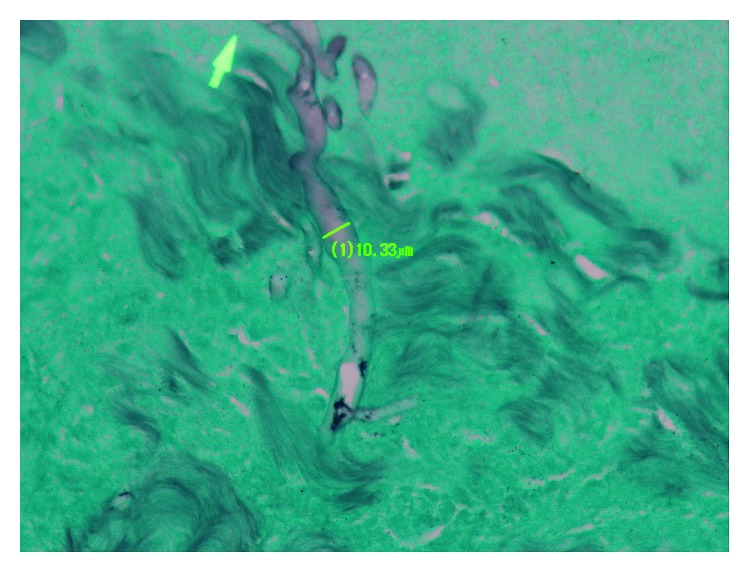
Methenamine silver stain showing mold with irregular nonseptate hyphae.

Pathology specimen of the gastrectomy identified an angioinvasive mold with irregular nonseptate hyphae visualized, consistent with invasive mucormycosis (Figure 1). Concomitantly, respiratory cultures grew *Aspergillus fumigatus*, and serum (1⟶3)-*β*-D-glucan was noted to be elevated to 294 pg/mL. On hospital day 27, he was started on liposomal amphotericin B and voriconazole to treat the dual fungal infections while awaiting identification of the mucormycete. Since tissue was not sent for culture, paraffin-embedded formalin fixed tissue sections were sent to the University of Washington Medical Center for molecular identification. Fungal DNA amplification using a 28S (D1/D2 region) primer set followed by sequencing was used to identify the isolate as *Mycotypha microspora* (GenBank accession number MH680712) [5].

The patient was noted to have significant kidney injury on admission with worsening of his renal function on the dual antifungal regimen. After 20 days of amphotericin therapy in combination with voriconazole, the decision was made to switch to isavuconazole in an attempt to avoid permanent nephrotoxicity. On hospital day 65, after completing two weeks of isavuconazole, the level of serum (1⟶3)-*β*-D-glucan was 246 pg/mL, and a repeat CT of the chest showed an increase in numerous bilateral pulmonary nodules and tree-in-bud infiltrates, both consistent with progressive aspergillosis. This was concerning for failure of the isavuconazole therapy. The patient was subsequently switched to posaconazole combined with micafungin, which was continued for 5 weeks, after which monotherapy with posaconazole was continued. On hospital day 85, a repeat CT of the chest showed decreasing multilobar nodular opacities. This was correlated with a decreased serum (1⟶3)-*β*-D-glucan level of 63 pg/mL. The patient did not have recurrence of GI bleeding and was discharged on hospital day 98 on oral posaconazole with a plan to continue treatment indefinitely pending resolution of pulmonary findings on repeat imaging.

## 3. Discussion

Mucormycosis is a life-threatening infection caused by fungi of the order Mucorales, with the most common genera causing disease being *Rhizopus*, *Mucor,* and *Lichtheimia* [6]. The hallmark of this disease is tissue necrosis resulting from aggressive angioinvasion and subsequent thrombus formation. Pathogenesis is thought to be due to invasion of endothelial cells by the binding of a fungal ligand belonging to a family of spore coating (CotH) proteins to the host receptor glucose regulator protein 78 (GRP78). These CotH invasins are universally present in Mucorales and blocking their function has been shown to reduce the ability of the fungus to invade and injure endothelial cells in vitro, as well as to reduce disease severity in mice [7, 8]. Once angioinvasion has occurred, necrotic tissue restricts access of antifungals to the infected sites, prohibiting clearance and allowing for further hematogenous dissemination of the disease [9].

Immunocompromised patients are most susceptible to invasive mucormycosis, presenting with distinct clinical syndromes including rhino-orbital-cerebral, pulmonary, gastrointestinal, and widely disseminated disease. Gastrointestinal mucormycosis is exceedingly rare and can involve either superficial colonization of ulcerative lesions or fungal invasion into the mucosa, submucosa, and vessels. Invasive disease is often fatal and mostly described in patients receiving immunosuppressant medications for solid organ transplants. Diabetes, corticosteroid use, or prolonged neutropenia are predisposing risk factors. Clinical manifestations of invasive gastrointestinal mucormycosis include appendiceal, cecal, or an iliac mass, and patients with neutropenia may present with fever, typhlitis, and hematochezia. Presence on histology or isolation of the fungus from tissue demonstrates proof of invasive fungal disease [10, 11].

First isolated from the peel of *Citrus aurantium*, *Mycotypha microspora* is a mucormycete that belongs to the order Mucorales [3]. It has previously been reported only as a colonizer, and to our knowledge, there are no published reports of *Mycotypha microspora* as a cause of angioinvasive mucormycosis in humans. Our case is the first example of biopsy proven angioinvasive disease with this species.

We encountered a number of challenges with this case, the most crucial being the need to treat two simultaneous invasive fungal infections, gastrointestinal mucormycosis, and invasive pulmonary aspergillosis in this immunocompromised patient. Current guidelines recommend serum assays for (1⟶3)-*β*-D-glucan for the diagnosis of invasive aspergillosis in high-risk patients and triazoles for treatment, with voriconazole being the azole of choice [12]. In contrast, there are currently no randomized controlled trials assessing the efficacy of antifungal regimens for mucormycosis given the rarity of the disease. Amphotericin is the most reliably active antifungal agent against mucormycosis. The lipid formulations of amphotericin are better tolerated but still carry significant adverse effects, including nephrotoxicity. Our patient had acute kidney injury on admission and developed worsening renal failure during his hospitalization, which was the limiting factor in our choice of therapy. The concern for permanent renal failure prompted the consideration of alternate formulations that would target both fungal species. Recently, isavuconazole, a novel triazole with broad spectrum antifungal activity, was shown to be noninferior to voriconazole in the primary treatment of invasive mold disease [13]. Our patient was treated with 15 days of isavuconazole; however, radiographic evidence of disease progression and persistent elevation of (1⟶3)-*β*-D-glucan raised concern for worsening pulmonary aspergillosis while on this antifungal drug. Case reports have been published describing the development of invasive fungal infection while on isavuconazole for empiric and targeted therapy in patients with hematologic malignancies and those who have had stem cell transplants [14, 15]. Suspicion for breakthrough invasive fungal infection while on isavuconazole prompted the decision to change therapy to a combination regimen of posaconazole and micafungin.

There is limited prospective clinical data to support the use of two simultaneous antifungal agents in the treatment of invasive fungal infections. Animal studies of invasive aspergillosis in transiently neutropenic guinea pigs suggest that combination therapy with an echinocandin and an azole leads to a decrease in fungal burden in tissues, as compared to monotherapy controls [16]. A prospective, multicenter, observational study of organ transplant recipients with invasive aspergillosis showed that in those with renal failure, combination therapy with voriconazole and caspofungin was independently associated with an improved 90-day survival [17]. Finally, a randomized, double-blind, placebo-controlled multicenter trial in over 400 patients with hematologic malignancies and hematopoietic stem cell transplants showed a higher rate of survival in patients with invasive aspergillosis who were treated with a combination of voriconazole and anidulafungin [18]. Though limitations in power precluded definitive conclusions about superiority, these data in combination with the animal models suggest that combination regimens represent a viable alternative to preferred therapies that are failing or toxic.

When isolated in culture, more common species of Mucorales may be identified via morphology and biochemical testing; however, molecular identification via direct amplification and sequencing is required to diagnose infrequently isolated organisms [19]. We postulate that since molecular identification is not routine to most clinical laboratories, this may result in underrecognition of rare species. To our knowledge, this is the first case of a unique species that has not been previously recognized as causing invasive infection, as well as its successful eradication in an immunocompromised host with concurrent invasive pulmonary aspergillosis.

Management of the catastrophic gastrointestinal bleed with a gastrectomy resulted in the eradication of the mucormycosis and was ultimately curative for our patient. Surgical resection is critical for the definitive treatment of gastrointestinal infection, as mortality is much higher without it [20]. The treatment of his invasive aspergillosis was limited by the need to preserve his renal function, and combination therapy ultimately proved to be a valuable option in his case as demonstrated by improvement in imaging findings and downtrending serum markers.
